# Supplementary material for: EnzML: multi-label prediction of enzyme classes using InterPro signatures
Source: BMC Bioinformatics. 2012 Apr 25;13:61. doi: 10.1186/1471-2105-13-61 (PMC3483700; doi:10.1186/1471-2105-13-61)
Supplement: Addtional file 5 — The Java code to format the data files, evaluate and predict. The file enzml_java_code.tar.gz contains the Java code used to format database data to ARFF and XML formats, to execute cross and train-test (jackknife) evaluations and to record evaluation results to database. More information is included in the readme.txt file and the Javadoc files. The code can be used with a MySQL database. To use a different database software, other JDBC drivers might be required. [file 1471-2105-13-61-S5.gz › java_code/utils/doc/uk/ac/ed/inf/utils/database/class-use/TableRow.html]

Uses of Class uk.ac.ed.inf.utils.database.TableRow


---


|  |  |  |
| --- | --- | --- |
| **Overview** \| **Package** \| **Class** \| **Use** \| **Tree** \| **Deprecated** \| **Index** \| **Help** | |  |
| PREV   NEXT | **FRAMES**    **NO FRAMES**     **All Classes** |  |


---


## **Uses of Class uk.ac.ed.inf.utils.database.TableRow**

| Packages that use TableRow | |
| --- | --- |
| **test.database** |  |
| **uk.ac.ed.inf.utils.database** |  |

Uses of TableRow in test.database

| Methods in test.database that return TableRow | |
| --- | --- |
| `static TableRow` | `TableRowTest.getRowKo()` |
| `static TableRow` | `TableRowTest.getRowOk()` |

Uses of TableRow in uk.ac.ed.inf.utils.database

| Methods in uk.ac.ed.inf.utils.database that return TableRow | |
| --- | --- |
| `TableRow` | `TableReader.getRowValues(java.sql.ResultSet resultSet, int rowNumber)`             Get the nth row of a `ResultSet` |
| `TableRow` | `TableReader.getTableRowWhereEqual(java.lang.String column, java.lang.String value)`             Gets the \*first\* table row where the given column has exactly the given value |
| `TableRow` | `TableReader.getTableRowWhereLike(java.lang.String column, java.lang.String value)`             Gets the \*first\* table row where the given column has a value similar to the given value |

| Methods in uk.ac.ed.inf.utils.database with parameters of type TableRow | |
| --- | --- |
| `java.lang.String` | `TableWriter.getInsertSql(TableRow row)`             Returns the sql "insert into" statement for the row |
| `java.lang.Integer` | `TableWriter.insertReturnsKey(TableRow row)`             Executes an sql 'insert into' operation. |
| `java.lang.Integer` | `TableWriter.writeRowToTable(TableRow row)`             Insert the row values into the appropriate database table |

---


|  |  |  |
| --- | --- | --- |
| **Overview** \| **Package** \| **Class** \| **Use** \| **Tree** \| **Deprecated** \| **Index** \| **Help** | |  |
| PREV   NEXT | **FRAMES**    **NO FRAMES**     **All Classes** |  |


---
